# Automatic Transportation Mode Recognition on Smartphone Data Based on Deep Neural Networks

**DOI:** 10.3390/s20247228

**Published:** 2020-12-17

**Authors:** Francesco Delli Priscoli, Alessandro Giuseppi, Federico Lisi

**Affiliations:** Control and Management Engineering “Antonio Ruberti”, Department of Computer, University of Rome “La Sapienza”, Via Ariosto 25, 00185 Rome, Italy; dellipriscoli@diag.uniroma1.it (F.D.P.); lisi@diag.uniroma1.it (F.L.)

**Keywords:** transportation model recognition, machine learning, artificial neural networks

## Abstract

In the last few years, with the exponential diffusion of smartphones, services for turn-by-turn navigation have seen a surge in popularity. Current solutions available in the market allow the user to select via an interface the desired transportation mode, for which an optimal route is then computed. Automatically recognizing the transportation system that the user is travelling by allows to dynamically control, and consequently update, the route proposed to the user. Such a dynamic approach is an enabling technology for multi-modal transportation planners, in which the optimal path and its associated transportation solutions are updated in real-time based on data coming from (i) distributed sensors (e.g., smart traffic lights, road congestion sensors, etc.); (ii) service providers (e.g., car-sharing availability, bus waiting time, etc.); and (iii) the user’s own device, in compliance with the development of smart cities envisaged by the 5G architecture. In this paper, we present a series of Machine Learning approaches for real-time Transportation Mode Recognition and we report their performance difference in our field tests. Several Machine Learning-based classifiers, including Deep Neural Networks, built on both statistical feature extraction and raw data analysis are presented and compared in this paper; the result analysis also highlights which features are proven to be the most informative ones for the classification.

## 1. Introduction

This paper presents a series of Machine Learning-based classifiers for real-time solution of the Transportation Mode Recognition (TMR) problem, i.e., the correct classification of which transportation system the user is travelling with. Efficient TMR classifiers require to find an optimal trade-off between performances, in terms of classification accuracy, and the resource demands, in terms of both data collection and computational effort, from users. With the diffusion of smartphones, it is now reasonable to assume to utilize a small portion of the computation resources of the users’ own devices—or even the computational capabilities provided by distributed nodes according to the paradigm of edge computing [1]—for the provision of a service that may improve the quality of life. Current solutions for turn-by-turn navigation already rely on continuous monitoring of the device sensors; therefore, it is possible to envisage an additional, small, background service for TMR in order to significantly improve the quality of the proposed travelling path. The Machine Learning solutions presented in this paper can be divided into two types: (i) statistical feature extraction-based approaches; and (ii) raw data analysis-based ones. The former type of solutions requires an additional computational step, as the process of statistical signal analysis and feature extraction can start only after the raw data is gathered and thus delays the transportation mode recognition, while the latter can start directly after the data is gathered and is hence more suitable for a real-time solution. Furthermore, more data should be gathered for statistical feature extraction-based analysis, as preserving all the information contained in the raw data through the feature extraction process may not be trivial. For this reason, the statistical feature extraction-based approaches will be trained and tested on data gathered during a time window of 2 s, while the raw data analysis solutions require approximately one second of samples, without any significant computational overhead.

As it will be discussed in Section 2, the main characteristics of this paper with respect to the state of the art are the following:

- The design of several ad-hoc Machine Learning classifiers to solve the TMR problem in two different data pipeline approaches, involving either statistical feature extractions or raw data analysis.
- The design of a Convolutional Neural Network to analyse raw data, characterised by an ad hoc non-sequential architecture.
- The reduction of the time window over which the analysis of the classifiers is conducted to 1–2 s, opening the possibility of having a TMR functionality running in the background of navigation applications due to the limited battery consumption associated with the data collection.
- The inclusion in the TMR formulation of seven different transportation modes of heterogeneous natures, namely, car, motorbike, walk, tram, still, subway and bus.

An immediate benefit for the market from a reliable solution to the TMR problem could be a more accurate estimation of aggregated users flows for the public transportation companies, one of the starting points for the efficient planning of public transport [2]. By the analysis of such transportation flows, the companies responsible for public transportation systems could be able to better schedule and plan their vehicle routes, even dynamically and in real time, while also providing a data-supported analysis for their transportation system expansion to investors [3]. Currently, this analysis is often conducted on statistical data obtained from socio-economic factors [4], but the availability of a real-time information flows enables the optimization of the aggregated transportation flows, by means of a dynamic response to specific demands (e.g., increasing the number of busses during certain periods of time over the day/year, or re-planning their routes). This approach would lead to a tangible increase in public transport users’ experience and, as a by-product, to a significant increase in the cities’ welfare and to the attraction of more investments. Controlling these flows could also help traffic management departments in reducing road congestions, even during emergencies, by better assessing the traffic status. For instance, by dynamically adjusting the traffic light intervals (for example in response to an emergency situation or to a particular peak of traffic flow) could greatly reduce the average travel time and the number of red lights experienced by the average user/emergency vehicle, in line with current research trends for smart traffic flow management [5]. Additionally, the analysis of the real-time aggregated traffic flows for the various transportation modes could help the real-time estimation and prediction of pollution levels [6].

Telecommunication system operators may also be interested in TMR solutions, as the anonymized customer data analysis coming from the statistical studies may be integrated into solutions compliant with the paradigm of edge- and fog-computing [7] and offered as a service to interested traffic operators and third parties. Another advantage of integrating a TMR solution in an edge/fog environment is that each fog node (e.g., a smart traffic light) may conduct its analysis autonomously, using a classifier trained with data gathered nearby its location. The benefit of such a scheme would be twofold: on the one hand, the classifiers trained on local data would have to solve an instance of the TMR problem that requires less generalisation (e.g., with respect to road condition/material), on the other hand, the overall accuracy of such classifiers may be higher, as they have to solve specialised instances of the problem.

The remainder of the paper is organised as follows: Section 2 discusses the state-of-the-art for the TMR problem, and the innovations introduced by this work. Section 3 presents our dataset structure and the two different proposed workflows for the two types of classifiers presented. Section 4 details the proposed classifiers. Section 5 reports the field test results, while Section 6 draws the conclusions and highlights possible future works.

## 2. State-of-the-Art and Proposed Innovations

Implementing a real-time identification of TMR is a challenging problem since the recognition has to be completed quickly, analysing only the data gathered in a small amount of time. The interest in TMR has risen recently as, with the development of 5G networks, applications that mine information from distributed and connected devices “at the edge of the network” have concrete economical returns. In this regard, the University of Sussex and Huawei Ltd. recently presented the SHL dataset [8] and its related research challenge [9] to encourage new research in the field. As discussed in Section 3, this work utilises a dataset gathered with a different philosophy than SHL. Historically, the first TMR solutions utilised Global Positioning System (GPS), as in [10,11], to infer features such as travel velocity, acceleration and their variance. For the very nature of the problem, the most common solutions involved Machine Learning methods, such as Decision Trees and Support Vector Machines, that commonly require the pre-processing of the data in order to extract the relevant and informative features. Solutions based only on GPS data are still being developed, as presented in [12,13]; but, with the increasing diffusion of smartphones, the second generation of TMR solutions started exploiting the sensors built into the various personal devices commonly utilised by the travellers. In this regard, the work in [14] provided an interesting approach to TMR, completely reliant on the accelerometer data gathered by the user’s smartphone, based on the extraction of the acceleration-related features from the raw data; in turn, [15] and [16] combined data coming from the GPS and the accelerometer of the smartphone to develop a classifier based on Decision Tree, a Hidden Markov Model and Support Vector Machines. As was presented in [17], the addition of accelerometer-based data significantly improved the classification performances, proving that the TMR process benefits from the analysis of data coming from a simpler, but more available and precise, data source. Nevertheless, the diffusion of Deep Neural Networks solutions, which exploit their capability of automatically identifying features from the raw data, have been proposed, as in [13], in which a Convolutional Neural Network that analyses GPS data is presented. A similar approach is taken in [18], where a Convolutional Neural Network was used for the classification on pre-processed accelerometer data. Interesting results regarding the benefit that higher sampling frequencies bring to TMR classifiers are presented in [19], proving that a significant portion of information is contained in the higher frequencies of the sensor signals. More recent works focused on either the feature extraction process, i.e., data pre-processing for more informative features identification, as in [20], or in post-processing with multi-stage classifiers, as in [21]. Works as [22] enhanced the learning capabilities of the classifiers commonly implemented in the literature by presenting a solution based on “extreme learning machines”, a promising Neural Network architecture that shows great generalization capabilities and fast convergence times. Finally, in a recent publication [23], the authors proposed a solution to classify five different transportation modes, proposing also methods for data reduction, such as Principal Component Analysis and Recursive Feature Elimination. Interesting concerns regarding the battery usage of TMR solutions were studied in [24], where the authors chose to utilise only local data sources available in the smartphone, disregarding the GPS data, considered too power-demanding for being utilised by real users. The same approach led to the design of the Deep Recurrent Neural Network presented in [25], which exploits data coming from both the accelerometers and gyroscopes of a smartphone.

Our work focuses on real-time solutions and hence the time window considered is of 2 s or lower, where most of the work presented above require several seconds or even minutes of data gathering. This assumption makes the proposed solution a better fit for on-device or fog computing-based solutions, due to its lower computational complexity and data transfer requirements. An interesting study dealing with real-time, background TMR is [26], where the authors developed a classifier based on statistical analysis (ANOVA and *t*-test) of the collected acceleration data against the reference distribution they identified for the transportation modes considered. Our work does not rely on the identification of such a distribution as we decided to exploit the generalisation properties of Machine Learning classifiers.

Finally, the authors of [27] report a comparison between several classifiers, focusing also on the process of feature selection starting from the data gathered in a window of 1 s, as in some of our classification solutions.

In Table 1 we report in a graphical way a summary of the state-of-the-art analysis of this section, to better highlight the various characteristics of the considered works from the literature.

To encourage the development of new solutions, we decided to make available the data gathered by the authors, which represents a portion of the data utilised to train the algorithms of this work, in the dataset TMRDataSet2020 [28]. The dataset contains data for a total of 7 transportation modes (walk, car, motorbike, tram, still, bus and subway), which represent the classes that our TMR solution classifies. The rest of the paper presents a series of machine learning solutions to deal with real-time TMR, trained on our data, spacing from statistical feature extraction-based approaches inspired from other existing solutions from the literature to Deep Neural Networks that run the classification directly on the raw data. The statistical feature-based solution will be treated as a benchmark for the performance of the Deep Neural Networks, and the results of the two approaches will be compared and discussed in the following. The classifiers that were implemented for this study consist of the following:

- Random Forest [29], as it is an ensemble learning solution based on Decision Trees, which were found in several works in the literature and represent one of the most typical baseline benchmarking solution for Machine Learning classifiers [30].
- Support Vector Machines [31], as it is one of the most widely used Machine Learning solutions for classification and was utilized in several recent works for TMR.
- Feed-Forward Neural Networks [32], as they represent the simplest neural network architecture and provide a valid baseline for more specialized solutions.
- Recurrent Neural Networks [33,34], as they were designed for time series analysis, such as the sensor readings involved in the TMR problem.
- Convolutional Neural Networks [35], as their characteristics proved to be effective in dealing with complex tasks, such as image and video analysis.

## 3. Data and TMR Workflow Description

The dataset consists of entries structured as reported in Table 2, which reports the order of the sensor readings, sampled at a frequency of 50 Hz over at least 8 s and gathered by 18 users during their normal travel routine from home to our Department. Note that, for the nature and quantity of the gathered data, the classifiers developed in our tests are not expected to be able to directly generalise in any scenario as the TMR problem heavily varies depending on vehicle and road conditions. In fact, the common factor of the data sources (geographic proximity) reflects on the generalization ability of the classifiers, which will become more specialized on the characteristics of the local area. The developed solutions shall then be seen as designed for an edge-computing framework, in which a computing hub gathers and analyses the data sources in its proximity, or a personalized solution that adapts to its specific user. This approach is complementary to the one of the SHL dataset [8], as it collects hundreds of hours of data from its three users, so that the classifiers trained on it may generalise better over different road conditions. The 8 s of data recording were randomly distributed over the user’s travel, to improve the generalisation of the TMR solutions, in such a way that approximately 10 recordings were collected per hour. A total of 140 h of travel were analysed, roughly evenly distributed among the various considered transportation modes.

A total of seven transportation modes have been studied in this work: car, motorbike, walk, tram, still, subway and bus.

For the sake of generalisation, the smartphone was kept in the pocket, in hand or on the dashboard of the vehicle. For all measures, the standard Google Android coordinate system from the official documentation [36] was considered.

Being heavily dependent on the carrier availability, the “Signal Strength” field of Table 2 is not considered in the following analysis; however, it may contain some high-level information to help the classification (e.g., subway may be differentiated from train based on the average signal strength) that, in future works, may require further testing and investigations.

The inclusion of GPS data, i.e., of the Latitude and Longitude fields, may also provide an interesting contribution to post-processing solutions, as, depending on the physical coordinates of the analysed user, the confidence level of the various classification choices may be updated (for example, in a rural area the probability of being in a subway could be lowered to make the classification choice “car” easier). In the analysis below, we decided to neglect the information related to GPS positioning, as our solutions focus more on direct classification approaches without post-processing, relying mostly on data gathered by the accelerometer and the gyroscope and keeping only the speed value, measured by the Android system, as GPS-based feature. Note that the inclusion of location information may also significantly impact the power required for the data gathering process.

The basic information used by the algorithms described in the following sections are resultant acceleration, velocity and angular speed. Absolute GPS measures are not used for energy-saving reasons (Android’s speed measures have an update rate of 1 s, meaning that the GPS usage is reduced to the minimum), but also to avoid having the classifiers discern the transportation modes depending on the location of the measurements (having a limited number of students that volunteer for the data collection process, which gathered the data mostly on their home-university trips, the classification basis could have been heavily influenced by the location of their houses).

Signal and magnetic strength do not seem to be informative in our use case, as their inclusion did not lead to any significant performance improvement; on the contrary, in some cases, it reduced the accuracy of our solutions.

The starting point of most of the works available in the literature is to consider the resultant acceleration $a_{r}$, obtained from its measured components $a_{x},a_{y},a_{z}$ on the Cartesian axes as

(1) $$a_{r}=\sqrt{a_{X}^{2}+a_{y}^{2}+a_{z}^{2}}$$

**Figure 1:**
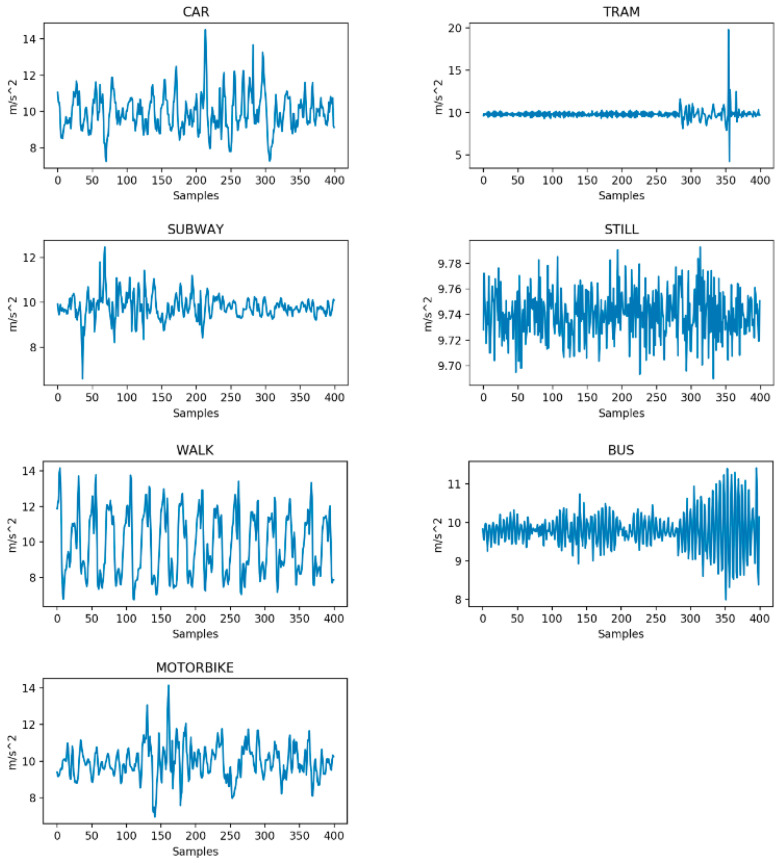
Resultant acceleration over 400 sampling times (8 s).

Figure 1 shows some examples of the resultant acceleration $a_{r}$ for the various considered transportation systems. By figure inspection, it is already clear that the various transportation modes differ in terms of amplitude and dominant frequency of their time-varying acceleration. However, inferring actual rules to discern among them is not a trivial task.

**Figure 2:**
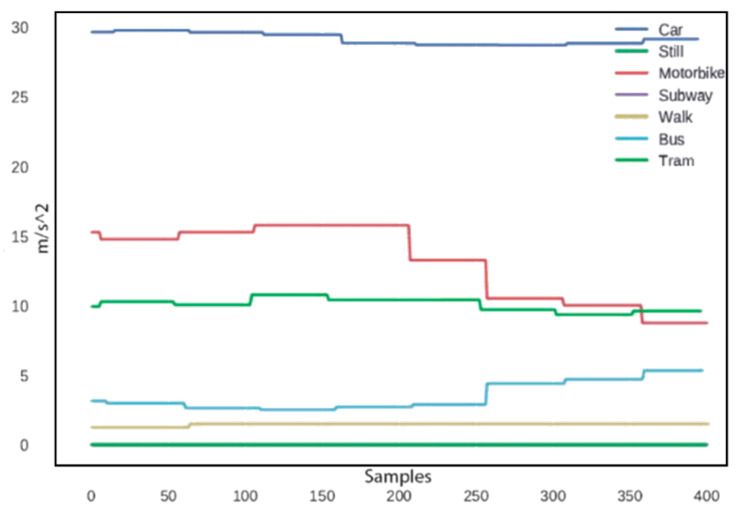
Velocity over 400 sampling times (8 s).

Regarding the velocity measures of the device, we noted that their evolutions are much smoother with respect to the acceleration profiles, as shown by the examples in Figure 2, which reports an example of the velocity profile for every transportation mode considered. This smoothness is due to the fact that the velocity measures returned by the Android API, derived from the GPS system data, are already averaged over 1 s. Note that since GPS is not accessible for the subway transportation mode, the corresponding velocity data are not available.

**Figure 3:**
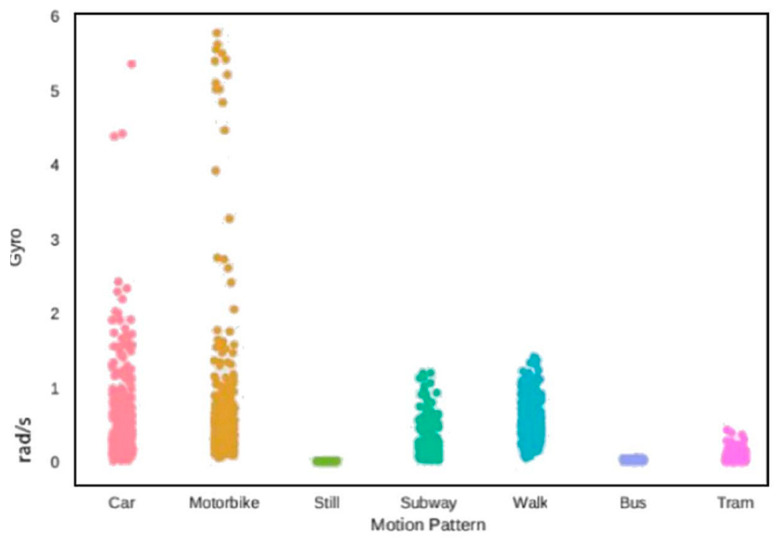
Module of the angular speed vector for the various transportation modes, during one sampling time.

Another interesting feature is how the phone orientation changes over time, i.e., its angular velocities. Figure 3 reports the angular velocity around the *x*-axis for the various transportation modes for a representative data sample and highlights that the motorbike has the largest variance, probably because even during small turns the driver and its motorbike have to partially tilt, while a considerable variance is also present in the walk and subway modes.

Similar to what was done for the acceleration, we will consider for the statistical feature analysis the norm of the vector of the angular velocities $\omega_{x},\omega_{y},\omega_{z}$:

(2) $$\omega =\sqrt{\omega_{X}^{2}+\omega_{y}^{2}+\omega_{z}^{2}}$$

**Figure 4:**
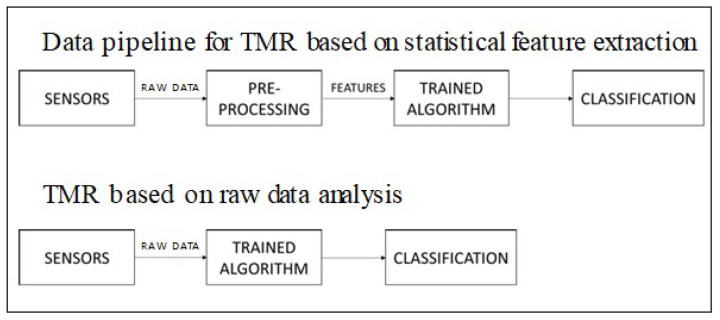
Functional flows for Transportation Mode Recognition (TMR).

In the following sections, we present the two workflows proposed for the design of the TMR solutions presented in this paper. In the first workflow, the classifier relies on statistical features that are extracted from the described raw data by means of a pre-processing stage, whereas in the second workflow the classifiers operate directly on the raw data. Figure 4 presents the schemes of the two TMR data pipelines.

### 3.1. TMR Based on Statistical Feature Extraction

The first proposed solution relies on a two-step approach: firstly, statistical features are extracted from the raw data collected by the device; secondly, a classification is performed on the basis of such features. This approach slows down the classification, but, if correctly done, can simplify the problem—in practice, the feature extraction step is a change of the coordinates on which the classification has to be solved to more convenient ones.

The extracted statistical features try to summarize the information contained in the various signals produced by the sensors. In particular, for all quantities we included their mean values and their minimum and maximum value over the sampling time, combined with their respective 95th and 5th percentile and standard deviation (std in the table). For acceleration and angular speed, we also considered kurtosis and skewness values, as well as the average and maximum values for the acceleration increase and decrease observed in the considered time window.

We considered that a natural set of additional statistical features to be explored lies in the Fourier domain, as was done in [6], as the vehicle motor vibrations translate into acceleration changes measured by the smartphone. To this aim, we conducted our analysis on the acceleration frequencies ranging from 1 Hz to 15 Hz.

All the considered statistical features are summarized in Table 3.

The 37 values of Table 3 are computed over 50 samples from the sensors, i.e., given the 50 Hz sampling time, over 1s. The various algorithms that rely on the extracted features, detailed in the following section of the paper, will be fed with the features extracted over two seconds—i.e., the statistical features are extracted from the concatenation of two vectors of 37 values each. This choice was driven by the trade-off between optimizing the classification performance (in terms of accuracy) and maintaining a low computational time.

### 3.2. TMR Based on Raw Data

Other classification solutions, such as Deep Neural Networks, are able to infer the correlation directly among the raw data, without the need of pre-processing it to extract additional features. In principle, these solutions are harder to train, but, in practice, their generalization properties are often more powerful, since Deep Learning solutions try to learn the most informative and suitable representation of the data during the training process [37].

Instead of providing the algorithms with the statistical features vectors of the 74 components (as with the feature-extraction based TMR solutions), the Deep Neural Networks considered in this work will be fed directly with the data coming from the sensors over a defined amount of sampling instants. In all our tests, the Deep Neural Networks (DeepNNs) were trained with the vectors of 64 raw samples, corresponding to 1.28 s of data at 50 Hz, almost halving the time-window dedicated to data collection. Together with the absence of data pre-processing, this reduction makes DeepNNs more suitable for real-time applications for TMR, also reducing significantly the amount of data to transmit to a remote server and the on-device computing requirements, thus lowering the power demanded by the application.

The following sections detail the various implemented solutions, briefly introducing their functioning logics and characteristics.

## 4. Classification Solutions for TMR

This section describes three TMR solutions based on statistical feature extraction, i.e., Random Forests, Feed-Forward Neural Networks and Recurrent Neural Networks, and two solutions based on raw data, i.e., a deep implementation of Feed-Forward Neural Networks and a Deep Convolutional Neural Network. The hyperparameters that characterize the various solutions employed for the comparison were determined, if not otherwise specified, following an iterative model selection procedure on the basis of a 5-fold cross-validation.

### 4.1. TMR Based on Feature Extraction

#### 4.1.1. Random Forest

Random Forests (RFs) [29] are ensemble methods that consider a group of “weak learners” as working together to form a “strong learner”, able to solve a complex problem. In RFs, the weak learners are Decision Trees [30], classifiers in which each class is represented by a leaf node of the tree, whereas each branch of the tree represents an attribute of the feature vector (e.g., speed > 3 m/s). The depth of the tree is defined as the maximum number of attribute checks that distance the root of the node (i.e., the starting point of the decision process) from the various leaves (i.e., the identified classes).

A Decision Tree is built iteratively from the analysis of the training dataset by selecting the criteria that better divides, in terms of average classification error, the considered population into different subsets. The process is repeated for the various subsets until a terminal condition is met, obtaining arbitrarily complex decision flows.

**Figure 5:**
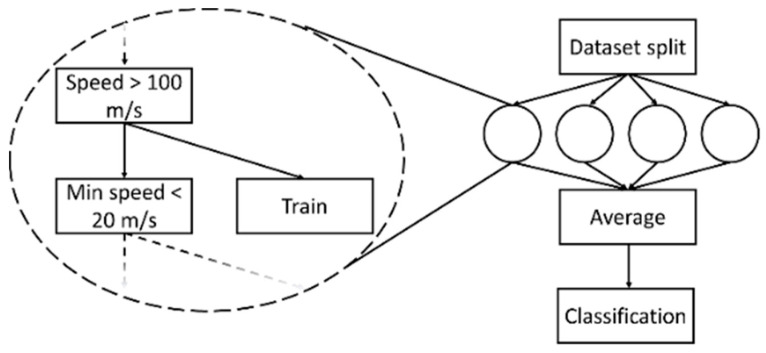
Random Forest high-level scheme.

The idea to build an RF classifier is to split the training dataset into multiple overlapping subsets and then to build a different Decision Tree for each of the subsets, potentially considering only a random subset of the available features. The classification results of the various trees are then averaged, and the most confident class is picked as the overall result of the classification. By so doing, the RF is expected to improve its generalization performance and to reduce overfitting problems with respect to a single Decision Tree. Moreover, the complexity of the Decision Trees can be kept down arbitrarily by fixing the maximum depth during their generation, making the overall decision problem complexity a tuning factor. Figure 5 reports a high-level scheme for RF description (the interested reader is referred to [38] and the bibliography therein to find a more detailed discussion of the training algorithms and fields of application). The RF used in this paper (see Section 4) is characterized by two parameters: number of trees, set to 1000, and their maximum depth, set to 14. The numerical values of these parameters were obtained by using a hyperparameter tuning technique based on the “out-of-bag (OOB) score” [39].

#### 4.1.2. Support Vector Machines

Support Vector Machines (SVMs) [31] are one of the most broadly used supervised learning solutions for classification, due to their ability to deal with nonlinear classification problems even in cases in which the dataset is heavily unbalanced.

In their most basic formulation, SVMs are a class of linear binary classifiers. Training an SVM means to find the hyperplane that separates the two classes available in the dataset in such a way that the Euclidean distance between the hyperplane and its closest elements, of both classes, is maximised. Utilising the so-called Kernel Trick [31], SVMs are able to efficiently find such a hyperplane in a higher dimensional representation of the original data, in which the two classes become linearly separable. The SVMs of this work use a radial basis function kernel.

#### 4.1.3. Feed-Forward Neural Network

Feed-Forward Neural Networks (FFNNs) [32] are a class of Artificial Neural Networks in which the connections between the various layers of neurons follow a single direction.

This type of neural networks represents the simplest multilayer solution, as an arbitrary number of hidden layers can be placed between the input and the output layers. The training process for such a network is usually based on the backpropagation algorithm [40], which updates the weights corresponding to the various synapsis between the neurons, and consequently their eventual activation, to minimize the error between the output provided by the network and the correct label.

For the feature extraction-based workflows, we consider a network with two hidden layers, fed with the extracted statistical features, having 74 neurons on the input layer, 512 and 32 sigmoidal neurons in the hidden layers, as well as 7 output neurons corresponding to the 7 identified transportation modes.

#### 4.1.4. Recurrent Neural Network

Working with time-dependent data, Recurrent Neural Networks (RNNs) represent a natural approach to explore. The architecture chosen for our testing consists in a multi-layer Long Short-Term Memory (LSTM) RNN [33,34]. The fundamental block of LSTM networks is the so-called memory cell, where the recurrence is given by the positive feedback on the cell state. Accessing the data stored in the cell state may improve the network’s decision, providing the ability to capture the long-term relation between different entries of the analysed time series. The update and access to this memory are regulated respectively by the input and the output gates, whose activation criteria are part of the training process itself. An additional gate, called the forget gate, is present to regulate when the state should be wiped. The RNN implemented in our testing was characterized by four hidden layers with respectively 512, 256, 128, and 32 cells.

### 4.2. TMR Based on Raw Data

#### 4.2.1. Deep Feed-Forward Neural Network

For the raw data-based workflows, we consider a Deep Feed-Forward Neural Network (DFFNN) with seven hidden layers, fed with raw data and having 60 sigmoidal neurons for the first three hidden layers and 40 for the other four hidden ones.

#### 4.2.2. Deep Convolutional Neural Network

Convolutional Neural Networks (CNN) [35] arose in popularity for their great performance in image analysis tasks. In principle this class of Neural Network is similar to the Feed-Forward one, with the main difference that each neuron has only local connectivity to the previous layer, meaning that it is connected only to a limited number of spatially close neurons. Furthermore, the neurons of the same layer share their weights and are often referred to as filters. Due to this weight sharing and the local connectivity, we can think of a convolutional layer as a single filter that moves over the input image (hence the name convolutional), producing an output that is a representation of the input and is referred to as feature map.

In more complex architectures, multiple filters can be run over the same input to capture different aspects of the starting features, each producing one corresponding feature map. We can visualize this solution as having multiple filters in the same layer and, correspondingly, multiple features maps as the output of the layer.

The main advantages of this type of architecture are the following ones:

1. The number of parameters to train is significantly reduced thanks to the fact that the weights are shared, with beneficial consequences in the speed of the training process and in avoiding overfitting.
2. The convolutional aspect of the moving filter, for image classification, removes the problem of the spatial location of the patterns to recognize [35] (e.g., a face is recognised independently on its absolute position in the picture). In our solution, passing to the network a matrix in which each row contains the seven raw measures (three from the accelerometer, three from the gyroscope and speed) at consequent sampling times, the spatial invariance property translates into time-invariance (e.g., a particular spike in acceleration may characterize the motorbike independently of where it appears in the time window, and, correspondingly, in the rows of the input matrix). For instance, if the pattern to be recognised is “a fast spike followed by an immediate drop in acceleration”, it is not of interest if this pattern appears at a given time or a few instants later. Given the 1.28 s over which the samples are collected, the input of the CNN is 64 × 7.
3. The more hidden level this architecture contains, the more complex patterns it can recognise, since we can think each feature map as a different representation of the starting features, with deeper layers capturing more complex concepts (as faces, objects, etc.) while the initial layers focus on simpler ones (edges, colours, etc.).

**Figure 6:**
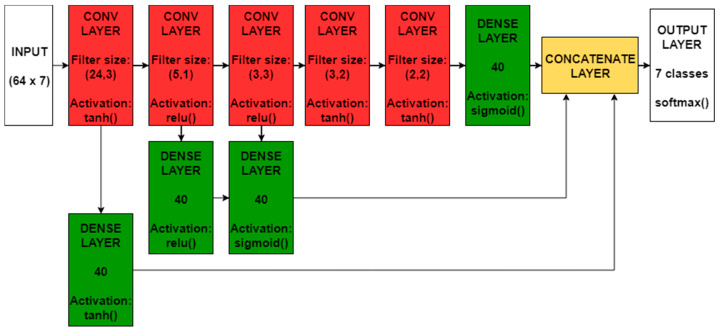
Deep architecture for real-time TMR.

The implemented neural network is a Deep Convolutional Neural Network (DeepCNN) characterized by the ad-hoc architecture reported in Figure 6, where a few convolutional layers with 32 filters each are connected to fully-connected feed-forward ones (referred to as Dense Layers in the Figure). The purpose of this kind of architecture is to collect information at various levels and forward it directly to the deeper layers. For this reason, the feed-forward layers are connected (after the concatenation of their outputs) to the final classification layer (Output Layer in the figure).

The information forwarding technique is conceptually similar to the idea behind Residual Networks [41], a ground-breaking solution for very deep neural networks that obtained stunning results in more complex classification tasks, such as image recognition. Connecting upper layers directly to the final one provided the best classification accuracy during our tests, and the architecture reported in Figure 6 was identified by means of model selection on the basis of a 10-fold cross-validation. Deeper models, such as fully Residual architectures, did not improve the model accuracy and proved to be more sensitive to noise and overfitting in general.

## 5. Results

This section reports the performance indicators for the trained algorithms in the validation procedure and in the field tests.

### 5.1. Validation

**Figure 7:**
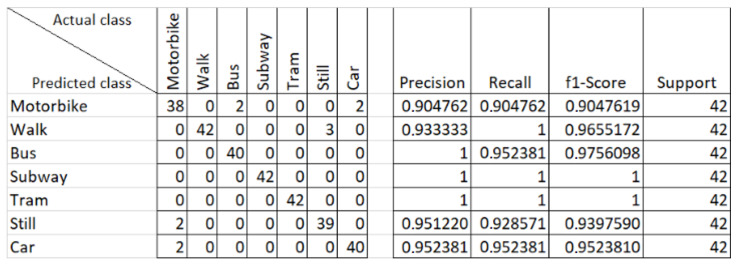
Confusion matrix for the Deep Convolutional Neural Network (DeepCNN).

The training process was firstly evaluated on the collected data by dedicating 25% of the collected data, randomly selected among the 8-s samples, to the validation set. Most of the hyperparameters presented so far were tuned over this validation. The results of the field tests validation, reported in the next section, are in line with the results of the tests performed on the validation sets; therefore, as an example of confusion matrices, we report at the end of the section only the ones relative to the DeepCNN, which was the best-performing solution in the validation phase. As reported in Figure 7, the performances of the DeepNN on the validation data are satisfactory, albeit we notice that the classification performs worse in identifying the motorbike class, which is confused with the car class and even the still class in some instances. Additionally, the still class was recognized a few times as the walk one.

### 5.2. Field Tests

The field test results were computed by the users by utilising a simple Android application developed for the purpose during their travel, in the same conditions in which the training data were collected. The Android application collects 2 s of data readings and forwards it to a remote server to run the various classifiers (the raw data-based ones only use the last 1.28 s of the received data), as would be the case in a solution that envisages the connection to a distributed computing node. We mention that an on-device solution could have been developed with modern mobile frameworks, such as Android Neural Network API (NNAPI) [42] and Basic Neural Network Subroutines (BNNS) [43], but the development of an on-device prototype was not in the scope of this work, as implementing and running all the presented classifiers on a smartphone would be extremely inefficient. The backend we developed on our server then returns and logs the transportation modes recognized by every classifier and each data sample, allowing a broad and fair comparison. A total of 100 tests were done for each transportation mode, using a total of two different Android phones, and the results are reported in the following tables. Firstly, we report in Table 4 the results regarding the statistical feature-based approaches.

During our testing, the most common classification errors were in the motorbike class, mostly confused with a car and still classed by all the proposed classifiers. It is interesting to note how the RF obtains the most uniform performances, surpassing the FFNN in the motorbike recognition; however, overall, the RNN obtains the best performance, recognizing particularly well the walk and still classes. The SVM is the only classifier that attains a perfect recognition on three transportation modes, but its overall performance is lowered due to its very low capacity in discerning a motorbike from a car. Notably, this classification mistake characterises all the statistical feature-based approaches and may be explained by the similarity of the sensor readings for the two classes, as is suggested from Figure 1 and Figure 3. We also mention that the statistical feature extraction process inevitably removes some information, so even if the two classes were distinguishable in the original raw data space, it is not surprising that the complexity of their recognition increased after the pre-processing.

Regarding the other solutions presented in this paper, Table 5 reports the results obtained by the analysis of the seven raw data measures without any pre-processing.

The overall best results are clearly obtained by the DeepCNN of Figure 6, while the DeepFFNN obtains performances that are also lower than the statistical feature-based RF and RNN classifiers. The generalization capabilities of the DeepFFNN were clearly offset by the increased complexity of the problem in the raw data space, which in turn required a more refined architecture as the one proposed for the DeepCNN. Even in the raw data space, the most challenging class remains the motorbike: during our test, the DeepCNN still sporadically confuses the bus and motorbike classes with cars, coherently with the reported results of the validation process. However, the recognition rate of the motorbike class is significantly higher with the raw data-based approaches (both the DeepFFNN and the DeepRNN ones), hinting that, probably, the extracted statistical features on which the other algorithms ran did not contain all the information needed to discern it from the other classes, which may be related to the quantities and relations not captured by the statistical analysis considered.

As already mentioned, besides the best performances obtained by the DeepCNN, another main advantage of the raw data-based solutions is that the collection of the needed entries requires 1.28 s (instead of the 2 s needed by the statistical feature-based solutions), enabling an almost real-time recognition.

## 6. Conclusions and Future Works

This work presented a series of different solutions for the real-time Transportation Mode Recognition problem, based on both statistical feature extraction methods and raw data analysis-based Deep Learning. The collected data, while being limited in size, represents a reasonable dataset gathered to train a locally specialised TMR classifier (e.g., for a traffic light or a road sensor). We reported field test results, highlighting how the analysis of the raw data performs overall better in terms of accuracy, which suggests that the feature extraction process removes a portion of the significant information.

Future work may include the implementation of more complex, multi-stage classification solutions, different Neural Network architectures, and the extension of the problem to more transportation modes.

## Figures and Tables

**Table 1:** State-of-the-art summary.

Reference	Number of Classes	Techniques																	Features					
		Bayesan Networks	Bivariate Movelets	Hidden Markov Models	Convolutional Deep NN	Decision Table\Rule Based	Decision Trees	Extreme Learning Machine	Feedforward Deep NN	Hierarchical Adaptive Boosting	K-Means Clustering	K-Nearest Neighbor	Naïve Bayes	Nearest Neighbor	Random Forest	Recurrent Deep NN	Statistical Analysis	Support Vector Machines	Accelerometer	Barometer	GPS	Gyroscope	Magnetometer	Raw
[10]	4																							
[11]	6																							
[12]	6																							
[13]	5																							
[14]	6																							
[15]	7																							
[16]	4																							
[17]	8																							
[18]	7																							
[19]	6																							
[20]	6																							
[21]	6																							
[22]	6																							
[23]	5																							
[24]	6																							
[25]	6																							
[26]	4																							
[27]	5																							
**This work**	7																							

**Table 2:** Complete data structure for the dataset utilised in this work.

Signal Strength		Speed	
Latitude		Longitude	
Acceleration-X	Acceleration-Y		Acceleration-Z
Gyroscope-X	Gyroscope-Y		Gyroscope-Z
Magnetometer-X	Magnetometer-Y		Magnetometer-Z

**Table 3:** The statistical features extracted in the pre-processing.

GPS/Speed	Mean speed, Max speed, Min speed, Speed std, Speed 5th Percentile, Speed 95th Percentile
Acceleration	Mean acc, Acc std, Max acc, Min acc, Acc 5th Percentile, Acc 95th Percentile, Acc kurtosis, Acc skewness, Acc components at {1,2,…,15} Hz, Acc increase avg, Acc decrease avg, Acc increase max, Acc decrease max
Gyroscope	Mean ω, ω std, ω kurtosis, ω skewness

**Table 4:** Feature-based approaches results.

	ACCURACY (%)			
Transportation Mode	RF	SVM	FFNN	RNN
Bus	86	93	89	93
Car	87	100	90	92
Motorbike	49	11	23	48
Still	88	52	80	99
Subway	90	72	81	92
Tram	84	100	83	93
Walk	86	100	90	99
(Average)	81.4	75.4	76.6	88.0

**Table 5:** Raw Data analysis approaches results.

	ACCURACY (%)	
Transportation Mode	DeepFFNN	DeepCNN
Bus	89	98
Car	85	96
Motorbike	28	96
Still	90	100
Subway	87	100
Tram	89	100
Walk	90	100
(Average)	79.7	98.6
